# ΔNp63 isoform-mediated β-defensin family up-regulation is associated with (lymph)angiogenesis and poor prognosis in patients with squamous cell carcinoma

**DOI:** 10.18632/oncotarget.1819

**Published:** 2014-03-21

**Authors:** Meggy Suarez-Carmona, Pascale Hubert, Arnaud Gonzalez, Anaelle Duray, Patrick Roncarati, Charlotte Erpicum, Jacques Boniver, Vincent Castronovo, Agnès Noel, Sven Saussez, Olivier Peulen, Philippe Delvenne, Michael Herfs

**Affiliations:** ^1^ Laboratory of Experimental Pathology, GIGA-Cancer, University of Liege, Liege, Belgium; ^2^ Laboratory of Tumor and Developmental Biology, GIGA-Cancer, University of Liege, Liege, Belgium; ^3^ Metastasis Research Laboratory, GIGA-Cancer, University of Liege, Liege, Belgium; ^4^ Laboratory of Anatomy, Faculty of Medicine and Pharmacy, University of Mons, Mons, Belgium

**Keywords:** p63, defensins, (lymph)angiogenesis, prognosis, squamous cell carcinoma

## Abstract

Beside a role in normal development/differentiation, high p63 immunoreactivity is also frequently observed in squamous cell carcinoma (SCC). Due to the complexity of the gene, the role of each p63 isotype in tumorigenesis is still confusing. Constitutively produced or induced in inflammatory conditions, human beta-defensins (HβDs) are cationic peptides involved in host defenses against bacteria, viruses and fungi. Here, we investigated both the role of p63 proteins in the regulation of HβDs and the implication of these antimicrobial peptides in tumor (lymph)angiogenesis. Thus, in contrast to TAp63 isotypes, we observed that ΔNp63 proteins (α, β, γ) induce HβD1, 2 and 4 expression. Similar results were observed in cancer tissues and cell lines. We next demonstrated that ΔNp63-overexpressing SCC are associated with both a poor prognosis and a high tumor vascularisation and lymphangiogenesis. Moreover, we showed that HβDs exert a chemotactic activity for (lymphatic) endothelial cells in a CCR6-dependent manner. The ability of HβDs to enhance (lymph)angiogenesis in vivo was also evaluated. We observed that HβDs increase the vessel number and induce a significant increase in relative vascular area compared to negative control. Taken together, the results of this study suggest that ΔNp63-regulated HβD could promote tumor (lymph)angiogenesis in SCC microenvironment.

## INTRODUCTION

Member of the p53 family, *TP63* gene gives rise to transcripts that encode either full-length isoforms containing an amino-transactivation (TA) domain (TAp63) or truncated isoforms that lacks this TA domain (ΔNp63). Both TA and ΔN transcripts undergo C-terminal alternative splicing to yield six further carboxyl-terminal isotypes (α, β, γ) [1]. In the last decade, the implication of p63 proteins in epithelial stratification [2], differentiation [3] and in the maintenance of the proliferative potential of epithelial stems cells [4] has been well established. In addition to their role in normal development and homeostasis, the large majority of squamous malignancies display p63 immunoreactivity suggesting that, similar to p53, p63 is also acting during tumorigenesis [5]. However, due to both the complexity of the gene and the lack of reliable antibodies for each individual isotype, the role of p63 in cancer is still controversial and subject to debate [6, 7]. Recent data suggested that *p63* could play a dual function. Indeed, several studies have highlighted the oncogenic potential of ΔNp63α [8-11]. In contrast, other data show that the *p63* gene, especially TAp63 isoforms, could act as a tumor suppressor [12-14], although *p63* is rarely mutated in human cancer in contrast to classic tumor suppressor genes.

Defensins are a family of small (2–6 kDa) cationic, antimicrobial peptides either constitutively secreted or induced in inflammatory conditions. Based on their amino acid sequence and pattern of disulfide bonding, mammalian defensins are classified into two main subfamilies: α and β defensins. Abundantly expressed by polynuclear neutrophils, α defensins were also isolated from subpopulations of macrophages and Paneth cells of the small intestine. To date, six human beta defensins (HβD1 to 6) have been discovered and cloned. Whereas HβD5 and HβD6 are specifically produced in the human epididymis, HβD1-4 are expressed by epithelial cells lining numerous organs (oral, nasal and epidermal mucosa, lungs, gastrointestinal and urogenital tracts) [15-17]. Through their direct antimicrobial activities, HβDs have emerged as important effectors of innate immunity [17]. Moreover, HβDs induce T cell and immature dendritic cell chemotaxis through chemokine receptor CCR6 and, therefore, might also link innate and adaptive immune responses [18-20]. Besides their role in the host defense, recent reports suggest that HβD expression could enhance tumor progression through unclear mechanisms [21]. By inducing dendritic cell and tumor-associated macrophage chemoattraction into cancerous lesions, it was proposed that HβDs could stimulate the production of tumor-promoting cytokines [22]. Moreover, *in vitro* data support that HβD2 could have some pro-angiogenic abilities [23].

The purpose of this study was to examine the regulation of HβD expression by p63 isoforms, as suggested in published microarray analyses [3, 24], and the implication of these small antimicrobial peptides in tumor vascularization and lymphangiogenesis. We showed that ΔNp63 proteins (α, β, γ) induce HβD1, 2 and 4 up-regulation whereas TAp63 isotypes do not modify HβD expression. These *in vitro* data were congruent with results obtained in cancer tissues [squamous cell carcinoma (SCC)]. Through a series of *in vitro* and *in vivo* experiments, we also demonstrated that ΔNp63-regulated HβDs are associated with tumor angiogenesis and lymphangiogenesis.

## RESULTS

### Positive association between ΔNp63 expression and HβD1, 2 and 4 levels in human keratinocytes and SCC cell lines

**Figure 1:**
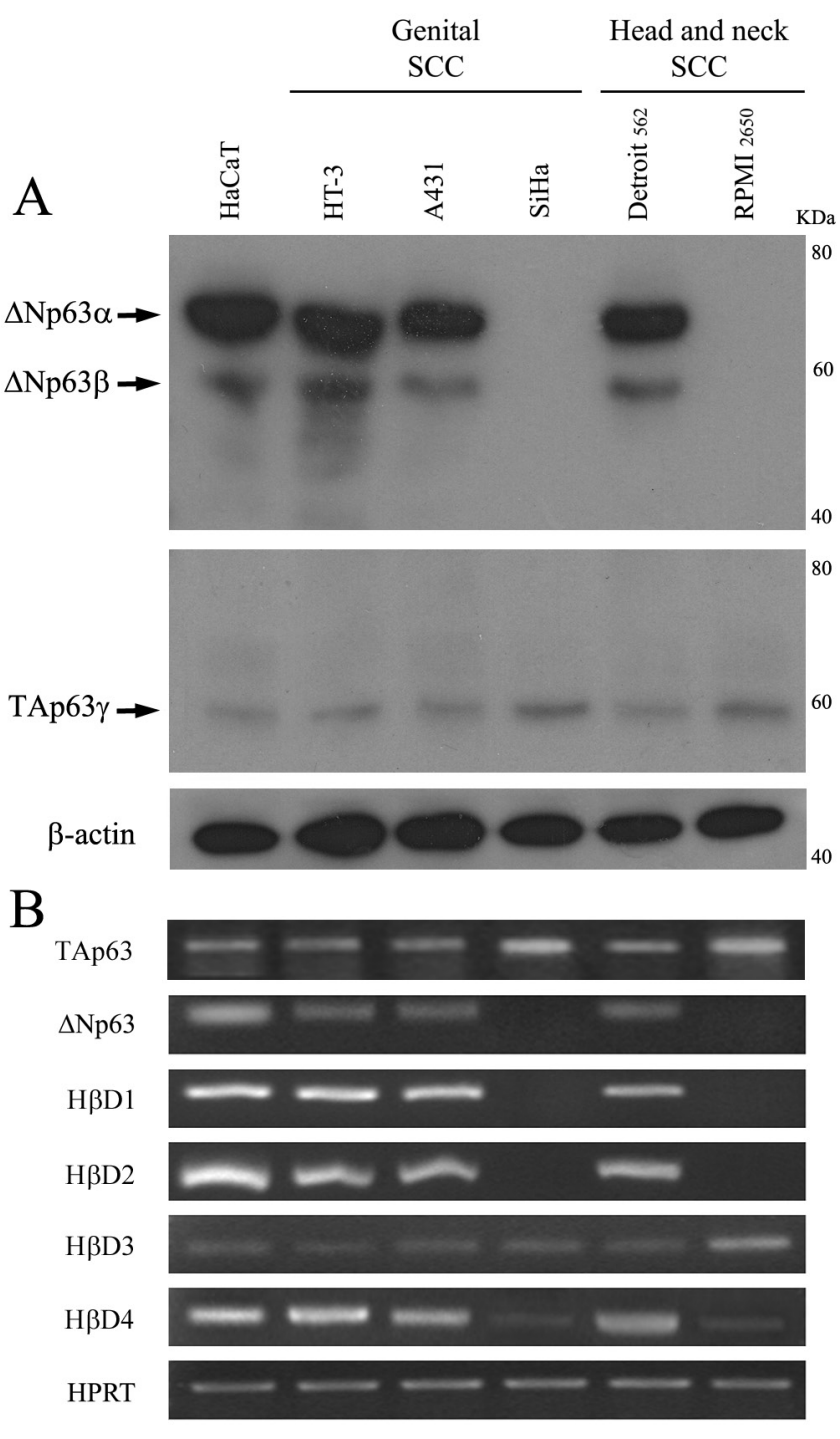
Expression of the p63 isoforms and HβDs in human normal keratinocytes and SCC cell lines A: Both ΔNp63 and TAp63 isotypes were detected by Western blot using anti-p40 (ΔN) and anti-TAp63 antibodies. B: RT PCR analysis of HβD1, HβD2, HβD3, HβD4, ΔNp63 and TAp63 isoform expression was performed on mRNA isolated from normal keratinocytes (HaCaT cells) and five human SCC cell lines. HPRT was used as controls for RNA loading. 10^5^ cells (from every analyzed cell line) were plated in six-well plates. When 60–70% confluence was reached, cells were lysed and subsequent Western Blot or RT PCR analyses were performed. The experimental procedures are extensively described in the “Materials and Methods” section. A representative experiment is shown of three independent experiments performed.

To determine the possible relationship between p63 isoforms and HβD family, we first analyzed their expression (Western blot and/or RT-PCR) in human normal keratinocytes (HaCaT) as well as in two head and neck (Detroit 562, RPMI 2650) and three genital (A431, HT-3, SiHa) SCC cell lines. As shown in Figure 1A-B, all these cells expressed TAp63. Regarding ΔNp63 expression, normal keratinocytes and several SCC cell lines (A431, HT-3, Detroit 562) exhibited extremely high protein and mRNA levels of ΔNp63 whereas SiHa and RPMI 2650 cells did not express this p63 isoform. Interestingly, in contrast to other cell lines, no or a weak mRNA expression of HβD1, 2 and 4 was observed in SiHa and RPMI 2650. No difference in HβD3 expression was detected between ΔNp63-positive and negative cells. Therefore, our data suggest that ΔNp63 could be involved in the regulation of HβD1, 2 and 4 expression.

### ΔNp63 isoforms regulate HβD1, 2 and 4 expression

**Figure 2:**
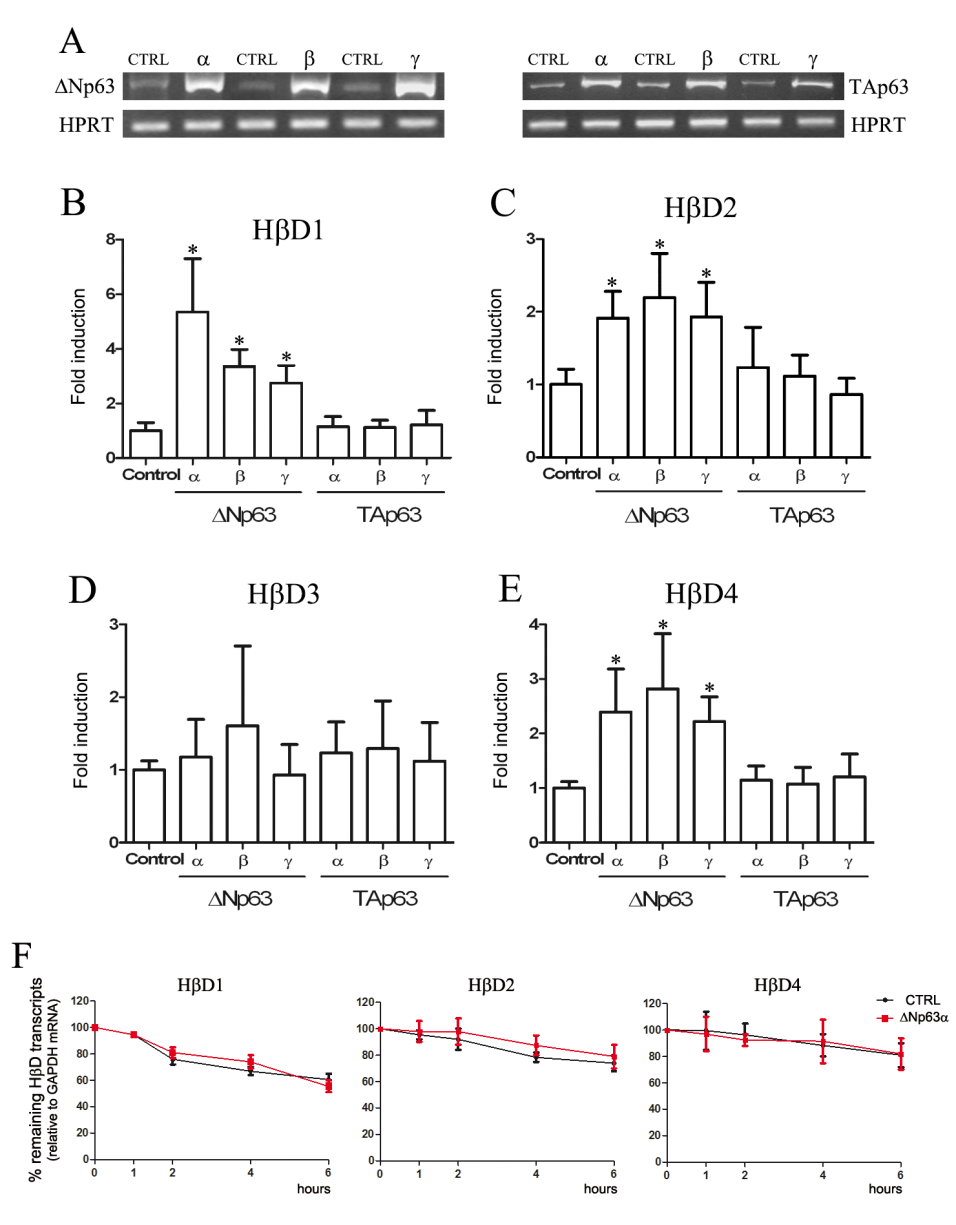
HβD1, HβD2 and HβD4 expression is up-regulated by ΔNp63 transfection A: cDNA corresponding to different p63 isotypes were transfected in HaCaT cells. Forty-eight hours after transfection, mRNAs were extracted and RT-PCR analyses were performed using primers specific for each p63 isoform. B-E: Real-time RT-PCR analysis of HβD1, HβD2, HβD3 and HβD4 expression were performed on mRNA isolated from HaCaT cells transiently transfected with the different p63 isoform cDNAs. At 48h after transfection, cells were collected for transcriptional analysis. The experimental procedures are extensively described in the “Materials and Methods” section. F: ΔNp63-transfected cells were treated with 5μg/ml actinomycin D 24h after transfection. mRNAs were isolated at the indicated times after actinomycin D application. Each real-time RT-PCR experiment was normalized to the amount of GAPDH mRNA from the same sample. Results are the means ± SD of four independent transfection experiments performed in duplicate. Asterisks indicate statistically significant differences (*P < 0.05).

To examine whether p63 proteins could regulate HβD expression, we transfected different p63 isoform cDNA sequences in normal keratinocytes and HT-3 SCC cell lines. p63 isoforms were upregulated at 24h (data not shown) and remained overexpressed until 48h (Figure 2A). HβD expression was assessed by real-time RT-PCR. Each experiment was normalized to the amount of HPRT mRNA from the same sample. As a control, cells were transfected with the corresponding empty vector. We showed that transient transfection of ΔNp63 isoform cDNAs (Figure 2B-E) for 48h significantly induce HβD1, 2 and 4 expression in HaCaT cells. No statistical difference in HβD3 expression was observed (Figure 2D). In contrast to ΔNp63 isoforms, TAp63α, β and γ did not alter the pattern of HβD expression (Figure 2B-E). No synergistic effect was detected when cells were simultaneously transfected with all ΔNp63 isoform cDNAs (data not shown). Similar results were observed with the cervical HT-3 SCC cell line (data not shown). In order to determine whether ΔNp63 isoforms influence HβD mRNA stability, we evaluated the rate of mRNA degradation. The RNA transcription activity was inhibited by actinomycin D and the mRNA level of HβD 1, 2 and 4 relative to GAPDH mRNA was then determined by quantitative real-time PCR. HaCaT cells were transiently transfected with ΔNp63α cDNA and, 24h after transfection, the cells were treated with 5μg/ml actinomycin D. The levels of HβD 1, 2 and 4 mRNA transcripts were determined at 1, 2, 4 and 6h following actinomycin D addition. Compared to control cells (empty vector), no significant difference of the rate of HβD 1, 2 and 4 decay was observed in ΔNp63α-transfected cells (Figure 2F). Similar results were obtained with other ΔNp63 isotypes (data not shown). These data suggest that ΔNp63 do not modulate HβD transcript levels via an increased mRNA stability.

**Figure 3:**
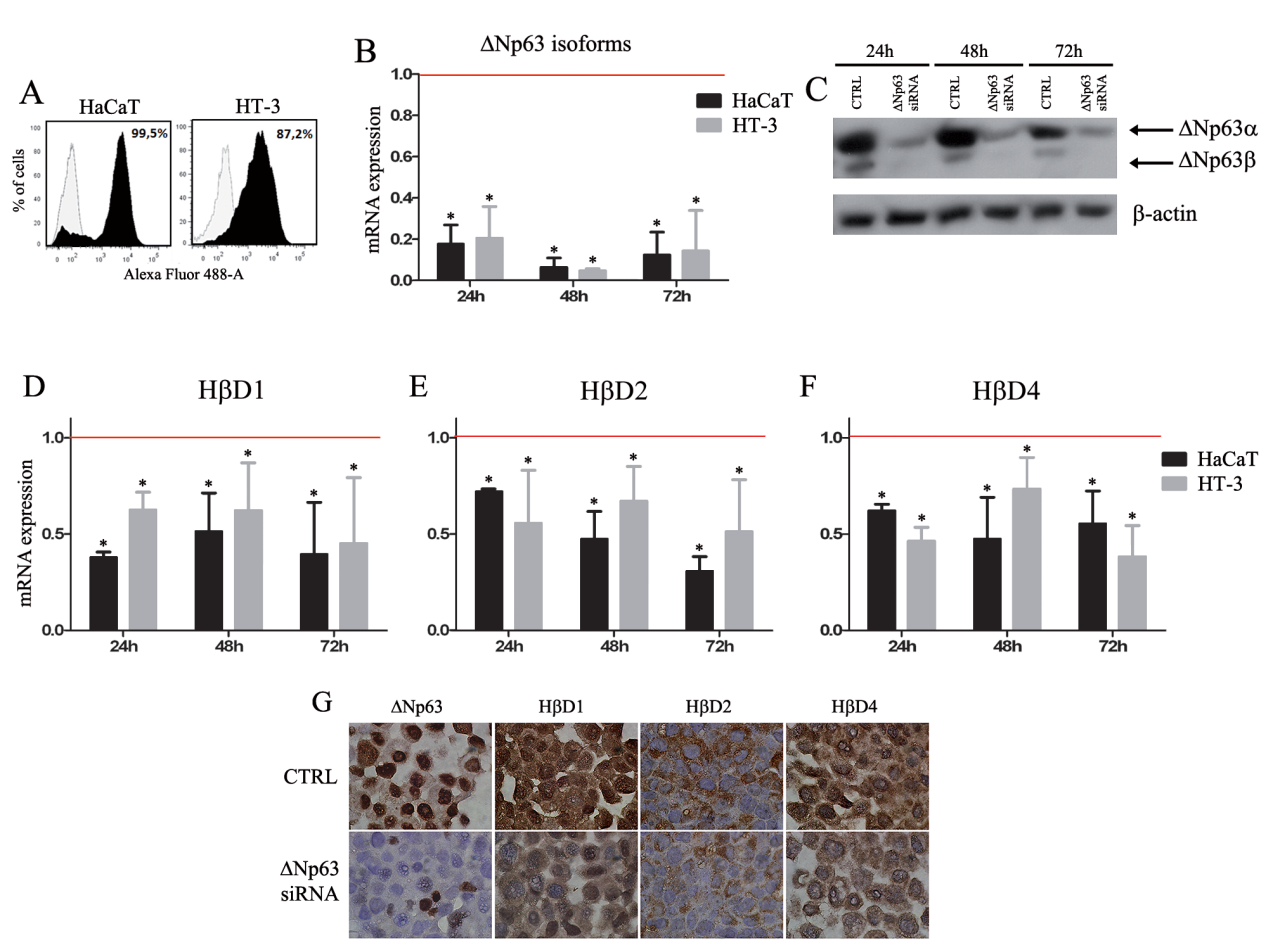
ΔNp63 silencing reduces HβD1, HβD2 and HβD4 expression in both normal keratinocytes and cancer cells A: First, siRNA transfection efficiency was assessed by flow cytometry using a Alexa Fluor 488-labeled control siRNA. B: Real-time RT-PCR analyses of ΔNp63 isoform expression were performed on mRNA isolated from ΔNp63 siRNA-transfected HaCaT and HT-3 cells. As a control, these cell lines were transfected with a siRNA which does not match to any sequence in the human genome. Each experiment was normalized to the amount of HPRT mRNA from the same sample. Results are the means ± SD of four independent transfection experiments performed in duplicate. C: ΔNp63 protein levels were also determined by Western blot in the ΔNp63-silenced HaCaT cells compared with the control cells. A representative experiment is shown of three independent experiments performed. D-F: Real-time RT-PCR analyses of HβD1, HβD2 and HβD4 expression were performed on mRNA isolated from ΔNp63-silenced HaCaT and HT-3 cells. Each real-time RT-PCR experiment was normalized to the amount of HPRT mRNA from the same sample. Results are the means ± SD of four independent transfection experiments performed in duplicate. Red bars represent corresponding controls for each condition. Asterisks indicate statistically significant differences (*P < 0.05). G: The HβD protein level was also evaluated on HaCaT cells transfected or not with ΔNp63 siRNA. A reduced HβD1, 2 and 4 immunoreactivity was observed in ΔNp63-silenced cells.

Inversely, we investigated the effect of an inhibition of ΔNp63 on HβD1, 2 and 4 expression using RNA interference strategy both in normal keratinocytes and cancer cells. siRNA transfection efficiency was assessed by flow cytometry (Figure 3A). ΔNp63 silencing efficiency (24, 48 and 72h after transfection) was analyzed by real-time RT-PCR (Figure 3B), Western blot (Figure 3C) and immunohistochemistry (Figure 3G). As a control, cells were transfected with a siRNA which does not match to any sequence in the human genome. Results indicated that ΔNp63 silencing significantly reduce HβD1, 2 and 4 expression in normal and cancer cells (at least 30% decrease compared to siRNA control-transfected cells) (Figure 3D-F). This downregulation of HβD1, 2 and 4 in ΔNp63-silenced cells was also observed at the protein level (Figure 3G).

### ΔNp63 immunoreactivity is associated with high levels of HβD1, 2 and 4 expression in cervical and head and neck SCC

**Figure 4:**
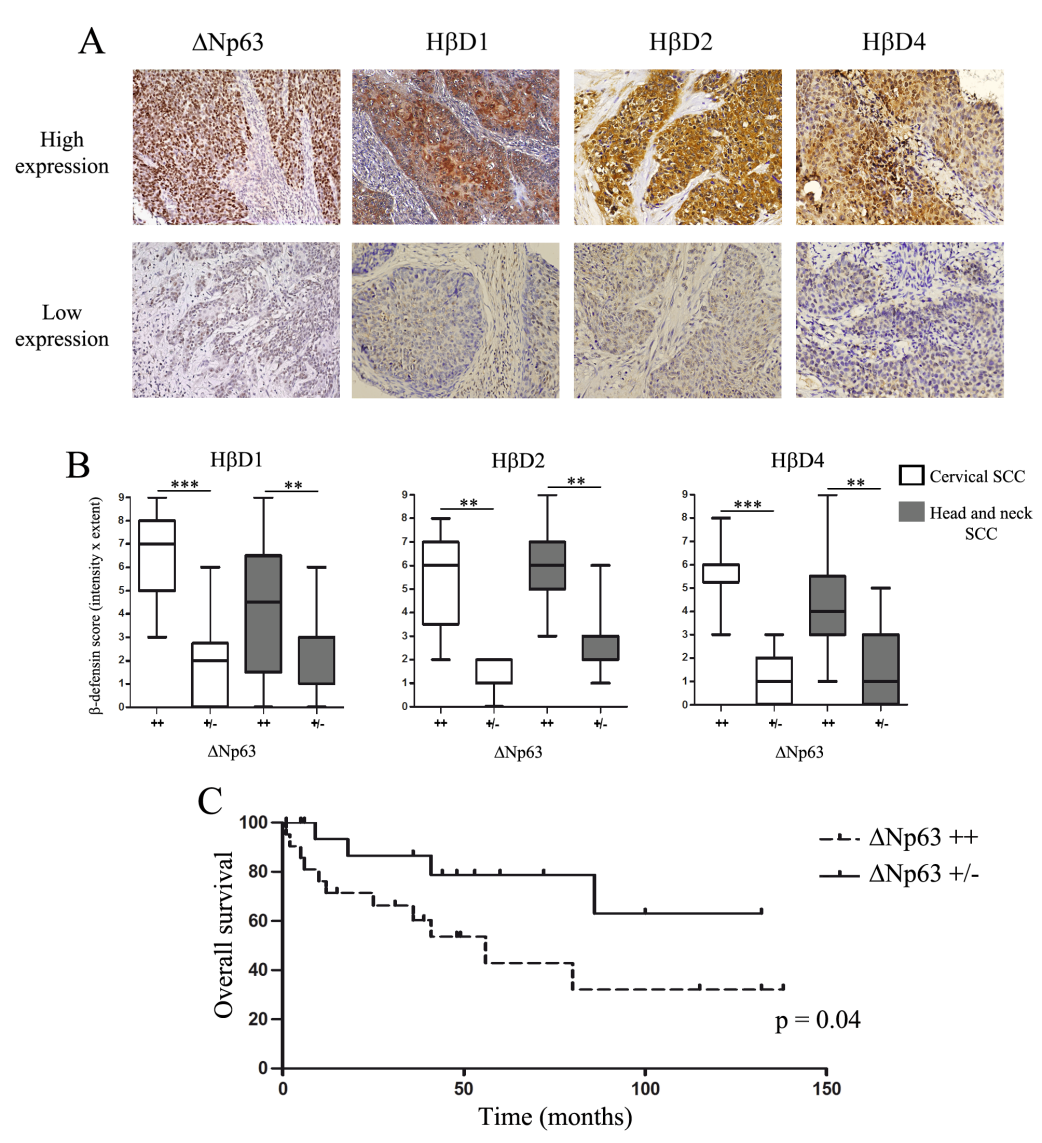
High HβD1, 2 and 4 immunoreactivity is observed in human SCC overexpressing ΔNp63 A: HβD1, HβD2, HβD4 and ΔNp63 expression in paraffin-embedded sections of human SCC specimens was assessed by immunohistochemistry. We observed variable degrees of HβD1, HβD2, HβD4 and ΔNp63 expression. B: Semiquantitative evaluation of HβD1, HβD2 and HβD4 expression in 18 cervical and 39 head and neck SCC specimens. The tissue samples were classified into two groups according to ΔNp63 immunoreactivity (high: ++, low: +/-). Asterisks indicate statistically significant differences (**P < 0.01, ***P < 0.001). Original magnifications: X200. C: Kaplan-Meier curve (overall survival) for patients with head and neck SCC expressing low (n= 18) or high (n=21) levels of ΔNp63.

By immunohistochemistry, the expression of HβD1, 2, 4 and ΔNp63 was then investigated in 18 cervical and 39 head and neck SCC specimens (Figure 4). Positive staining for ΔNp63 was observed in 54 tissue samples (94.7%). However, variable degrees of nuclear ΔNp63 expression were detected (Figure 4A). High expression of ΔNp63 (score >3) was observed in 9 (50%) cases of cervical SCC and in 21 (53.8%) cases of head and neck SCC. Furthermore, we analyzed HβD1, 2 and 4 expression in all these tissue specimens. These peptides were distributed in the cytoplasm of neoplastic cells. A nuclear HβD1 staining was also observed in 16 cases (28.1%) (Supplemental Figure 1). We showed that tumors with a highly positive ΔNp63 immunoreactivity were significantly associated with a global up-regulation of HβD1, 2 and 4 (Figure 4B). Indeed, a co-expression of ΔNp63 and hβDs was observed in numerous serial sections of SCC (Supplemental Figure 2). A similar association between hβD immunoreactivity and ΔNp63 expression was observed in normal squamous epithelia (Supplemental Figure 3). A Spearman correlation between ΔNp63 and HβD scores was also observed both in patients with cervical and head and neck SCC (Supplemental Figure 4). These results support the involvement of ΔNp63 in the regulation of HβD1, 2 and 4 in cancer tissues.

### Prognostic value of ΔNp63 expression in head and neck SCC samples

Due to the very few number of clinical data available [6 (33%) out of 18 patients], the prognostic value of cervical SCC samples was not analyzed. The follow-up data of the head and neck SCC patients for up to 138 months were used to evaluate the impact of ΔNp63 expression on overall survival. The series of head and neck SCC specimens presented in this study was composed of men (n=33) and women (n=6) from 40 to 79 years of age. Eighteen (46.2%) of these cancers were infected by high-risk HPV. Importantly, 27 (69.2%) and 12 (30.7%) of these patients were respectively active smokers or drinkers. None of these clinicopathological features were correlated with a high ΔNp63 expression (Table 1). During the follow-up period, 11 of 21 (52.3%) patients in the “ΔNp63++” group and 4 of 18 (22.2%) patients in the “ΔNp63+/-” group died. Based on Kaplan-Meier survival analysis, overall survival for patients with ΔNp63-overexpressing SCC was significantly decreased compared to that for individuals with cancers displaying a weakly positive ΔNp63 immunoreactivity (Figure 4C).

### HβD1, 2 and 4 enhance tumor angiogenesis in ΔNp63-positive SCC through promoting endothelial cells migration

**Figure 5:**
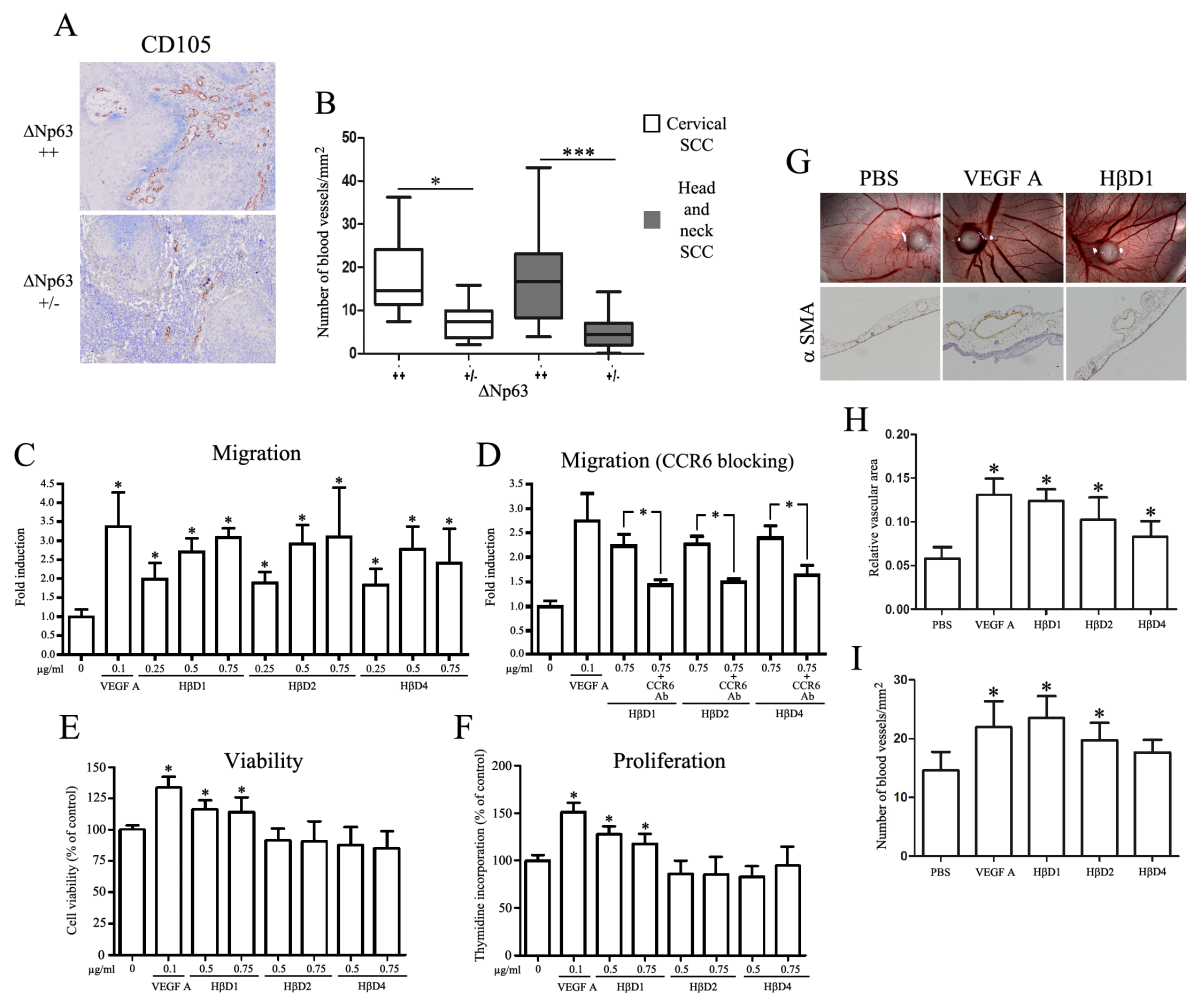
ΔNp63-regulated HβDs enhance endothelial cell migration A: CD105 immunostaining showing the neoformed blood vessel density in high and low ΔNp63-expressing SCC. Increased counts of CD105^+^ blood vessels were detected in high ΔNp63-expressing cervical and head and neck SCC when compared to tumors with a weakly positive ΔNp63 immunoreactivity (B). The average number of blood vessels per mm^2^ was measured as described in the “Materials and Methods” section. Influence of HβDs on endothelial cell migration in a Boyden Chamber assay (C), viability (E) and proliferation (F). The HβDs-related migratory ability of blood vessel cells was also measured with a CCR6-blocking antibody (D). PBS and VEGF-A were used as negative and positive control, respectively. Data are presented as the means ± standard deviation of four different experiments. G: Enhancement of angiogenesis by HβDs in the CAM assay. Representative images of alpha-SMA positive-blood vessels are shown. Quantification of blood vessels has been performed by computerized image analysis. (H) Relative vascular area and (I) number of blood vessels per mm^2^ were determined. Asterisks indicate statistically significant differences (*P < 0.05).

We next analyzed the blood vessel density in high and low ΔNp63-expressing cervical and head and neck SCC using anti-CD105 antibody. Also called endoglin, this accessory protein of the transforming growth factor beta receptor system is highly expressed on vascular endothelial cells [25]. Through CD105 immunolabeling on tumor sections, the average number of neoformed blood vessels per mm^2^ was evaluated by image analysis. As shown in Figure 5A-B, we observed that ΔNp63-overexpressing SCC are associated with a significantly higher tumor vascularisation compared to cancers displaying a weakly positive ΔNp63 immunoreactivity.

**Table 1:** Variable analysis for ΔNp63 expression in head and neck SCC

	High ΔNp63	Low ΔNp63	
			P
Clinical factors			
Age (y), median (SD)	58.1 (8.4)	57.6 (10.1)	
Sex			0,6674
Male	17	16	
Female	4	2	
Smoker			1
yes	15	12	
no	6	6	
Drinker			1
yes	6	6	
no	15	12	
Primary site			0,5279
Oral cavity	8	9	
Oropharynx	13	9	
T stage			0,7424
T1-T2	14	13	
T3-T4	7	5	
HPV DNA			0,738
positive	9	9	
negative	10	7	
			

In order to explore the ΔNp63-regulated HβD impact on the endothelial cell recruitment, Boyden Chamber migration assays were performed. As expected, VEGF-A elicited a considerable HUVEC migration, rising to 340% of the control (Figure 5C). A significant chemotactic activity of HβD1, 2 and 4 was observed for endothelial cells at a concentration as low as 0.25 μg/ml. When employed at a 0.5 μg/ml concentration, these three HβDs elicited a chemotactic effect similar to that exerted by VEGF-A, the positive control (Figure 5C). Furthermore, the HβD1, 2 and 4-dependent chemotaxis was similar when 0.5 μg/ml or higher concentrations were used (Figure 5C). We next investigated the involvement of chemokine receptor CCR6 on the capacity of vascular endothelial cells to migrate toward HβDs. Interestingly, pretreatment of the endothelial cells with a CCR6-blocking antibody partially abrogated the migration induced by HβDs (Figure 5D). We also observed that, similar to VEGF-A, HβD1 promoted endothelial cell proliferation/viability (Figure 5E-F). In contrast, cell growth was not significantly modified when HβD2 and 4 were used. The angiogenic activity of HβDs *in vivo* was then investigated using the CAM assay (Figure 5G). On day 10, there was a high density of blood vessels within and around the methylcellulose sponge in CAMs exposed to VEGF-A and HβDs. Each CAM section was immunostained for alpha SMA, a marker whose expression is relatively restricted to vascular smooth muscle cells. We observed that both VEGF-A and HβDs induce a significant increase in relative vascular area compared to negative control (PBS) (Figure 5H). This higher blood vessel area observed in the presence of VEGF-A and HβDs relied on an increased vessel number (Figure 5I). Confirming the implication of HβDs in angiogenesis, an association between HβD expression level (mainly HβD1 and 2) and the density of blood vessels in both cervical and head and neck tumor specimens was observed (Supplemental Figure 5). Although we fail to reach a statistical significance in SCC tissues with HβD4, our data suggest that these peptides play a relevant role in tumor angiogenesis.

### ΔNp63-regulated HβDs senhance lymphangiogenesis by inducing lymphatic endothelial cell chemotaxis

**Figure6:**
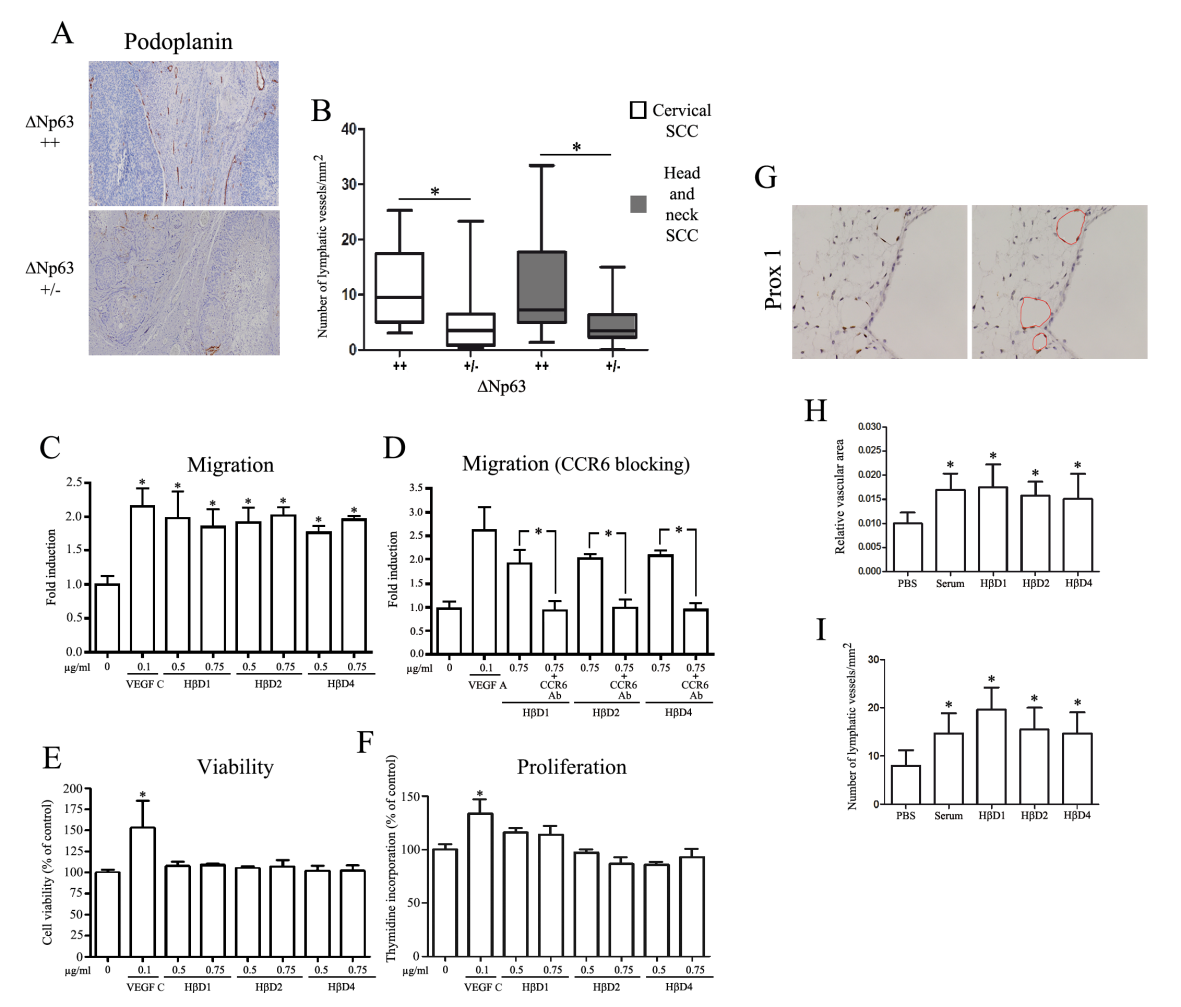
ΔNp63-regulated HβDs promote lymphangiogenesis A: Anti-Podoplanin antibody was used to determine the lymphatic vessel density in high and low ΔNp63-expressing SCC. Increased counts of podoplanin^+^ lymphatic vessels were observed in high ΔNp63-expressing cervical and head and neck SCC when compared to neoplastic lesions with a weakly positive ΔNp63 immunoreactivity (B). The average number of lymphatic vessels per mm^2^ was determined. Influence of HβDs on lymphatic endothelial cell migration (C), viability (E) and proliferation (F) was analyzed as described in the Materials and Methods section. Boyden chamber assay of lymphatic endothelial cells with CCR6-blocking antibody was also performed (D). PBS and VEGF-C were used as negative and positive control, respectively. Data are presented as the means ± standard deviation of four different experiments. G: Enhancement of lymphangiogenesis by HβDs in the CAM assay. Representative images of Prox1 positive-blood vessels are shown. Quantification of lymphatic vessels has been performed by computerized image analysis. (H) Relative vascular area and (I) number of lymphatic vessels per mm^2^ were determined. Asterisks indicate statistically significant differences (*P < 0.05).

The lymphatic vasculature was finally investigated in cervical and head and neck SCC, by evaluating the average number of podoplanin^+^ vessels per mm^2^. Significantly increased density of lymphatic vessels was detected in high ΔNp63-expressing SCC when compared to tumors with a weakly positive ΔNp63 immunoreactivity (Figure 6A-B).

The influence of ΔNp63-regulated HβDs on chemotactic migration of lymphatic endothelial cells was then assessed using a Boyden chamber assay. As shown in Figure 6C, a significant increased migration of immortalized (hTERT-LEC) lymphatic endothelial cells was observed in the presence of both VEGF-C (positive control) and HβD1, 2, 4 compared to negative control. Moreover, the chemotactic activity of HβDs on lymphatic endothelial cells was similar when we used 0.5 μg/ml or higher concentrations (Figure 6C). Similar results were obtained with normal cells (HMVEC) (Supplemental Figure 6). HβD-dependent enhanced migration was totally abrogated by addition of a CCR6-blocking antibody (Figure 6D). Regarding cell growth and viability, no difference was observed when lymphatic endothelial cells were treated with HβDs 1, 2 or 4 (Figure 6E-F). The lymphangiogenic activity of HβDs *in vivo* was then investigated using the CAM assay. Numerous vascular structures, lined by alpha SMA-negative endothelial cells, were detectable on CAM sections. Immunohistochemical staining for the lymphatic biomarker Prox-1 confirmed the lymphatic nature of these vessels (Figure 6G). Image analysis was used to quantify CAM lymphangiogenic response from optical microscopy observation (Prox-1 staining). We demonstrated that both serum (positive control) and HβDs induce a significant increase in relative lymphatic vascular area compared to PBS (Figure 6H). This increased lymphatic vessel area was relied on a higher vessel number (Figure 6I). Similar to angiogenesis, HβD-overexpressing tumor specimens were associated with a higher tumor lymphangiogenesis compared to cancers displaying a weakly positive HβD immunoreactivity (Supplemental Figure 7). Collectively, our results support the implication of HβDs in lymphangiogenesis.

## DISCUSSION

Whatever the tumor location (cervix, head and neck, esophagus…), the overall survival rate for SCC is low, largely due to the capacity of cancer cells to disseminate via blood and/or lymphatic circulations. The formation of new blood vessels is well-known to play a key role during the tumor growth and metastasis processes [26]. Similarly, accumulating evidence suggests that lymphangiogenesis also promotes the tumor progression and regional lymph node involvement is both early sign of metastasis and one of the strongest poor prognostic indicators. Understanding the interplay between cancer cells and (lymph)angiogenesis could define new molecular targets that might prevent the very initial stage of tumor spreading from the primary site and, therefore, increase patient survival [27]. However, the molecular and cellular basis of blood and lymphatic abnormalities associated with cancers remains unclear and the subject of numerous investigations.

HβDs are antimicrobial peptides produced primarily by epithelial cells. Recently, several studies have showed the implication of HβDs in immune cell chemotaxis, activation as well as in wound healing suggesting that the collective effects of these small peptides extend well beyond their antiviral/bacterial activities [21]. Moreover, reports have analyzed the expression of HβDs in normal and (pre)neoplastic tissues at mRNA and protein levels and showed that HβD-1 and 2 were particularly expressed in well differentiated oral SCC [28]. Although pro-inflammatory cytokines, bacterial products and TGF-β have been shown to induce HβD upregulation, transcription factors and associated signaling pathways that regulate the expression of HβDs still remain unknown. Furthermore, these data suggest that HβD expression could be regulated by multiple factors and be cell or tissue-type dependent [21].

In the current study, upon examination of several human SCC cell lines, we found a clear association between ΔNp63 and HβD-1, 2 and 4 expression. No difference in terms of HβD3 expression was observed between ΔNp63-positive and negative cells. In order to demonstrate this relationship between p63 and HβDs, we transfected p63 isotype cDNAs in normal and SCC cells and demonstrated that, in contrast to TAp63 isoform, ΔNp63α, β and γ increase HβD expression at the mRNA levels. This HβD upregulation was not related to an increased HβD mRNA stability. Inversely, a decrease of HβD-1, 2 and 4 expression following ΔNp63 silencing was shown in HaCaT and HT-3 cells. By immunohistochemistry, we demonstrated that this downregulation of HβD1, 2 and 4 in ΔNp63-silenced cells was also observed at the protein level. We tried to confirm all these results by Western blot analysis. Numerous protocols were used. However, similarly to other studies and probably because of the small size of these peptides (2–6 kDa), it was impossible to detect HβDs, excepted when 0.5 or 1μg of recombinant proteins (positive control) was used. In agreement with our results and according to published microarray analyses, HβD1 and 4 were two genes highly down-regulated (3 to 10 fold) after disruption of p63 expression in several cancer cell lines [24]. These data were obtained using a siRNA targeting all p63 transcripts.

To evaluate the association between HβD-1, 2 and 4 and ΔNp63 isoform expression in human SCC specimens, immunohistochemical analyses were performed. A significant increase in HβD1, 2 and 4 immunoreactivity was observed when ΔNp63 was overexpressed. In addition, ΔNp63 and HβD scores were correlated both in cervical and head and neck SCC confirming our *in vitro* results. Previous data from our laboratory and others showed variable degrees of nuclear p63 expression in SCC tissues [29, 30]. Furthermore, marked overexpression of ΔNp63 has been recently associated with increased proliferation, radiation resistance and unfavorable outcome in the context of several cancers [9, 31-33]. Although ΔNp63 may reduce apoptosis and promote cell proliferation, the implication of ΔNp63 isoforms in soluble factor expression and cancer microenvironment has not been extensively explored. We demonstrated that ΔNp63-regulated HβDs stimulate the migration of (lymphatic) endothelial cells in a CCR6-dependent manner which may explain partially the increased density of blood and lymphatic vessels observed in high ΔNp63-expressing SCC. Beside HβD upregulation, *in vitro* data showed that ΔNp63 overexpression increases secretion of Interleukin 6, 8 and VEGF-A which could also promote angiogenesis in SCC microenvironment [34-35]. Regarding CCR6, the requirement of this chemokine receptor in HβD-dependent enhanced migration was, however, higher in lymphatic cells suggesting that these peptides could promote blood vascular cell chemotaxis via an alternative receptor. The *in vitro* results showing an implication of HβDs in (lymph)angiogenesis were confirmed using the *in vivo* CAM assay. In contrast to chemoattraction, HβDs display a limited effect on viability and proliferation of endothelial cells. In agreement with our results, RÖhrl and collaborators recently showed an increased vascularization in mouse β defensin 14 overexpressing tumors. These authors also found that this enhanced angiogenesis is reduced in CCR6 knockout mice, indicating the requirement of CCR6 expression on mouse endothelial cells [36]. This chemokine receptor is also involved in HβDs-mediated human macrophage and mast cell chemoattraction.

Finally, the impact of ΔNp63 expression on overall survival was evaluated. Overall survival was significantly higher for patients with low ΔNp63 expression compared with those displaying a high expression for this p63 isoform. In agreement with our results, data reported that the overexpression of p63 in oral SCC was associated with poor radiation response and shorter survival [31, 32]. However, in contrast to our data, these studies did not reveal a potential mechanism and used a pan-antibody targeting all p63 transcripts; thus different p63 isoforms were not analyzed separately.

In conclusion, we demonstrated that ΔNp63 isoforms affect HβD expression in human SCC leading to increased blood and lymphatic vessel density in the tumor microenvironment and indirectly to a poor prognosis. Therefore, a treatment strategy aimed at reducing the adverse effects of HβDs may be effective at reducing metastasis dissemination and/or recurrence after surgery.

## MATERIALS AND METHODS

### Patients and tissue samples

Fifty-seven paraffin-embedded specimens of SCC [18 cervical SCC (mean age: 47 ± 9 years) and 39 head and neck SCC (33 men and 6 women; mean age: 58 ± 9 years)] were obtained from Pathology archives (University Hospital Center of Liege or Mons in the period between 2002 and 2010). Ten cases of normal ectocervical squamous epithelium were also retrieved. All cases were re-examined by a pathologist to confirm the diagnosis. Clinicopathological features were available for head and neck SCC patients (Table 1). These tissue samples were collected at the Tissue Bank of the University of Liege. The protocol was approved by the Ethics Committee of the University Hospital of Liege.

### Primary cells, cell lines and cell culture media

Normal human umbilical vein endothelial cells (HUVECs) and human microvascular endothelial cells (HMVEC) were purchased from Lonza (Verviers, Belgium) and were grown in MCDB 131 medium (Gibco-Invitrogen, Carlsbad, CA, USA) supplemented with 10% fetal bovine serum, 2mM glutamine, endothelial cell growth supplement (12 ng/ml; BD Biosciences, Bedford, MA) and 2.5 mg/ml heparin. Immortalized lymphatic endothelial cells (hTERT-LEC) were cultured in EGM2-MV (Lonza) supplemented with 5% fetal bovine serum and glutamine. Immortalized human keratinocytes (HaCaT cells) and three genital SCC cell lines (A431, HT-3, SiHa) were grown in a 3:1 mixture of DMEM (Gibco) and Ham's F12 (Gibco) containing 10% fetal calf serum (FCS) and supplied with 1% non-essential amino acid (Gibco), 1% sodium pyruvate (Gibco) and 0.5% penicillin-streptamycin (Gibco). Two head and neck SCC cell lines (Detroit 562, RPMI 2650) were maintained in MEM (Gibco) containing 10% FCS and supplemented with 1% L-glutamine (Gibco). All the cell lines were incubated until a 60–70% confluence was reached.

### Immunohistochemistry

Immunohistochemical analysis of paraffin-embedded specimens was performed as previously described [29, 37, 38]. Antibodies anti-ΔNp63 (anti-p40; Calbiochem, Gibbstown, NJ, USA), anti-HβD1 (Biologo, Kronshagen, Germany), anti-HβD2 (Abcam, Cambridge, MA, USA), anti-HβD4 (Abcam), anti-CD105 (Thermo Scientific, Waltham, MA, USA), anti-Podoplanin (Clone D2-40, Dako, Glostrup, Denmark), anti-alpha Smooth muscle actin (SMA) (Abcam) and anti-Prox1 (ReliaTech GmbH, Wolfenbuettel, Germany) were used for the primary reaction. Immunoperoxidase staining was performed using the Envision kit (Dako, Glostrup, Denmark) or the BrightVision Plus kit (Immunologic, Duiven, Netherlands) according to the supplier's recommendations. Positive cells were visualized using a 3, 3'-diaminobenzidine (DAB) substrate and the sections were counterstained with hematoxylin. A control IgG was used as negative control (Santa Cruz Biotechnology, Santa Cruz, CA, USA). To test the specificity of the HβD staining, the different anti-HβD antibodies were neutralized by the incubation with an excess of peptide. No immunoreactivity was observed in this condition.

### Immunostaining assessment

According to a protocol previously described [29, 37], two independent histopathologists evaluated the immunolabeled tissues by using a semi-quantitative score of the intensity and extent of the staining according to an arbitrary scale. For staining intensity, 0 represented samples in which the immunoreactivity was undetectable whereas 1, 2 and 3 denoted samples with, respectively, a low, moderate and strong staining. For staining extent, 0, 1, 2 and 3 represented samples in which the immunoreactivity was detectable, respectively, in <5%, 6–33%, 34–66% and >67% of the tumor cells. In order to provide a global score for each case, the results obtained with the two scales were multiplied, yielding a single scale of 0, +1, +2, +3, +4, +6 or +9. This scoring system was validated using a computerized image analysis system (CAS; Becton Dickinson, Erembodegen, Belgium). The biopsies were classified into two groups: high (score >3) or low (score ≤3) ΔNp63 expression. Moreover, both the relative vascular area and the density of blood (CD105^+^) and lymphatic (Podoplanin^+^ or Prox-1^+^) vessels in tumor microenvironment was quantified by computerized count (FSX 100 computerized image analysis system, Olympus, Hamburg, Germany), verified by manual counting and supervised by a histopathologist. The number of blood and lymphatic vessels was reported to the area around tumor cells yielding a count expressed as number of vessels/mm^2^. A similar computerized quantification method was used to quantify the number and the vessel density in lymph/angiogenic CAM images.

### Chicken chorioallantoic membrane (CAM) angiogenesis assay

On embryonic day 3, a window was open into the shell of fertilized chicken eggs. Four days later, methylcellulose 3 mm sponges saturated with 5 μg HβDs were laid onto the egg chorionallantoic membrane. VEGF-A (1.5 μg) (R and D systems, Minneapolis, MN, USA) or serum and PBS were used as positive and negative controls, respectively. Blood vessel density was evaluated and photographed on day 10. CAMs were then fixed (formalin) for 24 h, remove from the eggs and paraffin embedded using standard protocol. To identify blood and lymphatic vessels, anti-alpha SMA and anti-Prox1 were used, respectively. Thirteen CAMs were analyzed in each test group.

### RT-PCR and quantitative real-time PCR analysis

Total RNA was extracted (RNeasy mini kit, Qiagen, Valencia, CA, USA) and cDNA was generated by reverse transcription as previously described [39]. For quantitative real-time PCR experiments, 15 ng of cDNA were amplified in 25 μl of 1× SYBR-Green I qPCR master mix plus (Eurogentec, Seraing, Belgium), containing 200 or 300 nmol/L of each primer. Primer sequences were as follows: TAp63 forward, 5'-TGTATCCGCATGCAGGACT-3'; TAp63 reverse, 5'-CTGTGTTATAGGGACTGGTGGAC-3'; ΔNp63 forward, 5'-GAAAACAATGCCCAGACTCAA-3'; ΔNp63 reverse, 5'-TGCGCGTGGTCTGTGTTA-3'; HβD1 forward, 5'-GTCGCCATGAGAACTCCCTACC-3'; HβD1 reverse, 5'-CATTGCCCTCCACTGCTGAC-3'; HβD2 forward, 5'-CCAGTTCCTGAAATCCTGAG-3'; HβD2 reverse, 5'-CTCTGTAACAGGTGCCTTGA-3'; HβD3 forward, 5'-AGTGACCAAGCACACCTTTTCA-3'; HβD3 reverse, 5'-CCAAAAACAGGAAGAGCAAAGC-3'; HβD4 forward, 5'-CCTGTTACCTGCCTTAAGAGTG-3'; HβD4 reverse, 5'-GAATCCGCATCAGCCACAG-3'; HPRT reverse, 5'-GGTCCTTTTCACCAGCAAGCT-3'; HPRT forward, 5'-TGACACTGGCAAAACAATGCA-3'; GAPDH reverse, 5'- ACCAGGTGGTCTCCTCTGAC-3'; GAPDH forward, 5'- TGCTGTAGCCAAATTCGTTG-3'. Thermal cycling conditions were: 50 °C for 2 min, 95°C for 10 min, 40 cycles of denaturation at 95°C for 15 s and annealing/extension at 60 °C for 1 min. All the experiments were performed in triplicate, using the ABI-Prism 7700 Sequence Detection System (Applied Biosystems, Foster City, CA, USA) and negative controls (master mix without any cDNA or RNA) were added in each run. Each quantitative real-time PCR experiment was normalized to the amount of HPRT or GAPDH mRNA from the same sample. The acquired data were analyzed by Sequence Detector software, Version 1.9 (Applied Biosystems). As an additional control, PCR products were run on 1.8% agarose gels containing ethidium bromide and visualized with an UV transilluminator.

### Western blotting analysis

Cells were lysed in a buffer containing 50 mM TRIS pH 7,5, 300 mM NaCl, 1 mM EDTA, 1% Nonidet P-40 (Igepal CA-630) (Sigma, Saint Louis, USA), 1 mM PMSF (Sigma) and protease/phosphatase inhibitors (Roche, Bale, Switzerland). After quantification (BCA protein assay; Pierce, Rockford, USA), western blot analysis was performed as previously described [37]. Anti-β-actin (Sigma), anti-ΔNp63 (anti-p40, Calbiochem) and anti-TAp63 (Biolegend, San Diego, CA, USA) were used as primary antibodies.

### siRNA transfection and gene silencing

Small interfering RNA (siRNA) targeting specifically human ΔNp63 (5'-UGCCCAGACUCAAUUUAGU-3') was designed previously [29] and purchased from Eurogentec. The day before transfection, 10^5^ cells per well of a six-well plate were seeded in 2 ml of appropriate growth medium. For each experiment, 50 ng of siRNA duplexes were transfected with the TransFectin lipid reagent (Bio-Rad, Hercules, CA, USA) according to the supplier's recommendations. A siRNA which does not match to any sequence in the human genome (Eurogentec) was used as control. The transfection of an Alexa Fluor 488-labeled control siRNA (Eurogentec) was performed in order to analyze the percentage of cells with siRNA uptake.

### Transient transfections of p63 isoform cDNAs

pcDNA3 expression vectors (Invitrogen) encoding each p63 isoform were kindly provided by Dr. Caron de Fromentel (INSERM U590, Lyon, France). 1.25 x 10^5^ cells were plated in six-well plates. Twenty-four hours after plating, cells were transiently transfected with ExGene reagent (Fermentas, Burlington, Canada) (2 μg plasmid DNA, 9 μl ExGene in 200 μl of 150 mmol/L NaCl). As a control, cells were transfected with the corresponding empty vector. A control transfection condition using a plasmid encoding GFP (pEGFP-IRESpuro, Clontech, CA, USA) was performed in parallel to determine the transfection efficiency. All experiments were set up to obtain at least 60% of transfected cells.

### Boyden chamber migration assay

Chemotactic migration of HUVEC, HMVEC and hTERT-LEC were assessed using the Boyden chamber assay [18]. 3 x 10^4^ cells were suspended in serum-free medium supplemented with 0.1% BSA and placed in the upper compartment of a 48-well Boyden microchamber (Neuroprobe, Cabin John, MD, USA). For inhibitory experiments, blocking anti-CCR6 antibody (Clone 53103, R and D systems) was added 20 min at 37 °C before treatments were started. The lower compartment was filled with a VEGF-A (R and D systems) (0.1 μg/ml), VEGF-C (R and D systems) (0.1 μg/ml), ΗβD1 (PeproTech, Rocky Hill, NJ, USA) (0.25 to 0.75 μg/ml), ΗβD2 (PeproTech) (0.25 to 0.75 μg/ml) or ΗβD4(PeproTech) (0.25 to 0.75 μg/ml) solution, containing 0.1% BSA. After 24 h of incubation at 37 °C, the cells that had migrated to the underside of the 8μm gelatin-coated filter (Poretics Corp., Livermore, CA, USA) were fixed and stained with Diff Quick Stain set (Baxter Diagnostics AG, Düdingen, Switzerland). The upper side of the filter was scraped to remove residual non migrating cells. One random field was counted per well using an eyepiece with a calibrated grid to evaluate the number of fully migrated cells. Experiments were performed four times in sixplicate.

### Cell viability/proliferation

Cell viability and proliferation were determined by using the Alamar blue colorimetric-based assay (AbD Serotec, Dusseldorf, Germany) and the 3H-thymidine incorporation assay, respectively. Twenty four hours before stimulation with HβDs, 50,000 cells per well of a six well plate (viability) or 5,000 cells per well of a ninety-six well plate (proliferation) were seeded in appropriate growth medium. Both cell viability and 3H-thymidine uptake were measured after 4 days of culture. For the proliferation assay, the incorporated 3H-thymidine was normalized to the total number of cells.

### Statistical analysis

Statistical analysis was performed with Instat 3 software (Graph-Pad Software, San Diego, CA, USA). The statistical significance of the results was calculated by using a Student's *t* test (immunostaining) or a Mann-Whitney test (cell proliferation, migration, viability, quantitative real-time PCR). The correlations among the staining intensity of ΔNp63, HβD1, HβD2 and HβD4 were assessed using Spearman's correlation analysis. Categorical data from independent groups were compared using the Fisher's Exact Test. Overall survival was defined as the time from the date of registration to the date of death. Standard survival time analyses were performed using Kaplan–Meier curves. Differences were considered as statistically significant when *P* values were less than 0.05.

## SUPPLEMENTARY FIGURE


