# Supplementary material for: ΔNp63 isoform-mediated β-defensin family up-regulation is associated with (lymph)angiogenesis and poor prognosis in patients with squamous cell carcinoma
Source: Oncotarget. 2014 Mar 21;5(7):1856–68. doi: 10.18632/oncotarget.1819 (PMC4039122; doi:10.18632/oncotarget.1819)
Supplement: Supplementary file 1 [file oncotarget-05-1856-s001.pdf]

**ΔNp63 isoform-mediated β-defensin family up-regulation is associated with (lymph)angiogenesis and poor prognosis in patients with squamous cell carcinoma - Suarez-Carmona et al**

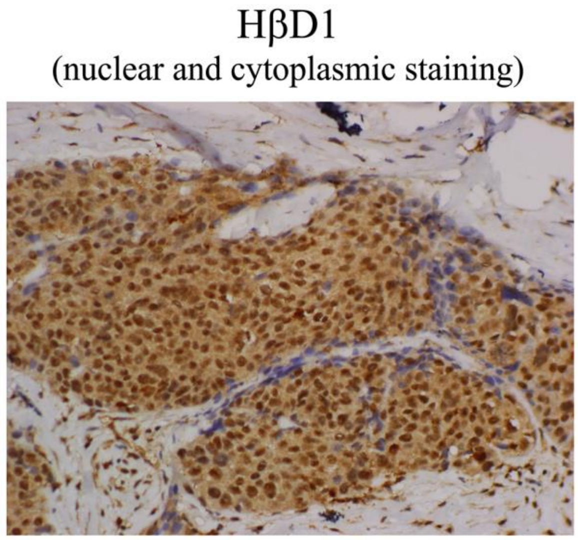


**Supplemental Figure 1.** Representative example of nuclear HβD1 immunostaining in SCC.

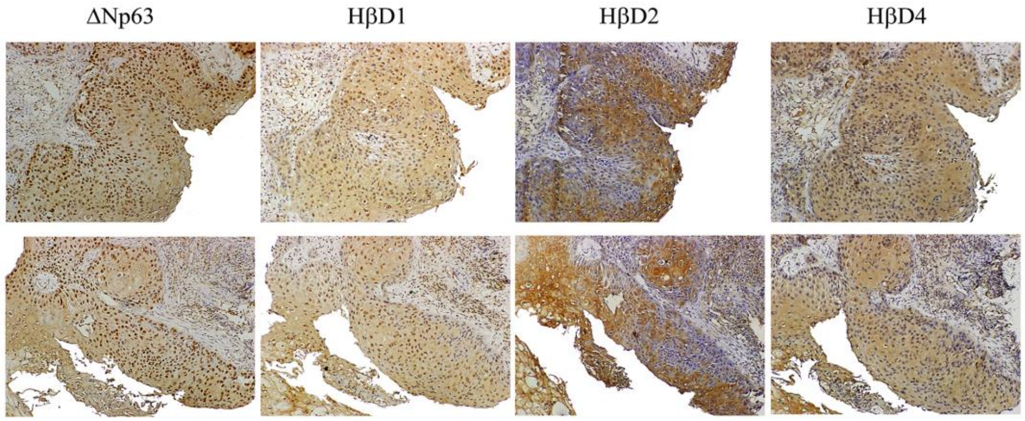


**Supplemental Figure 2.** Serial sections showing a co-expression of ΔNp63 and hβDs in cancer tissues.

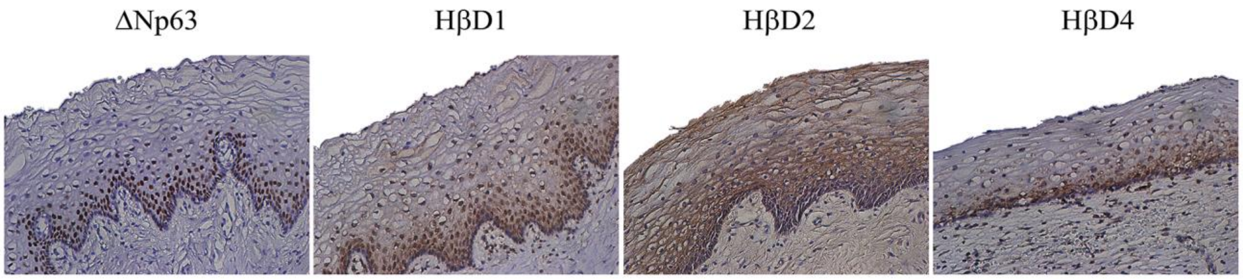


**Supplemental Figure 3.** Association between HβD immunoreactivity and ΔNp63 expression in normal ectocervical squamous epithelium.

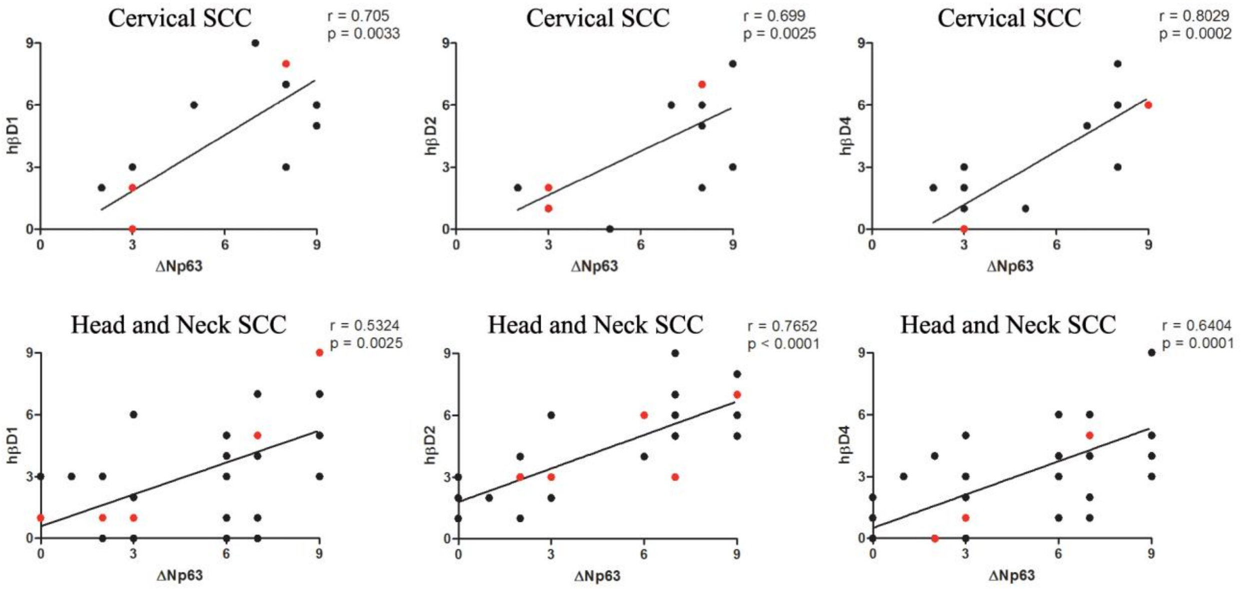


**Supplemental Figure 4.** Spearman's correlations between ΔNp63 and HβD expression in patients with cervical or head and neck SCC. *r* indicates the Spearman's correlation coefficient.

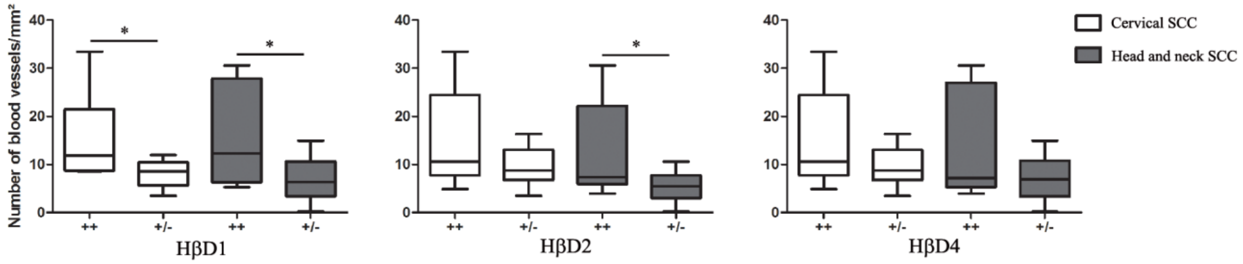


**Supplemental Figure 5.** Increased density of blood vessels was detected in high HβD-expressing cervical and head and neck SCC when compared to tumors with a weakly positive HβD immunoreactivity. The tissue specimens were classified into two groups: high (score >3) or low (score ≤3) HβD1, 2 or 4 expression. The density of blood vessels ($CD105^+$) in tumor microenvironment was quantified by computerized image analysis and verified by manual counting.

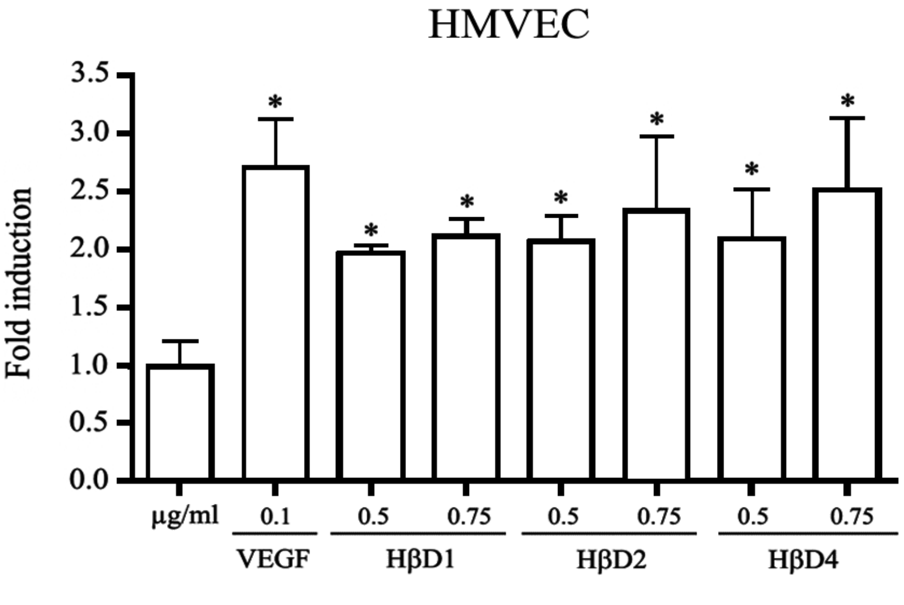


**Supplemental Figure 6.** Chemotactic activity of HβDs on human microvascular endothelial cells (HMVEC). PBS and VEGF-C were used as negative and positive control, respectively.

Results are the means ± standard deviation of six wells from three different experiments. Asterisks indicate statistically significant differences (*P < 0.05).

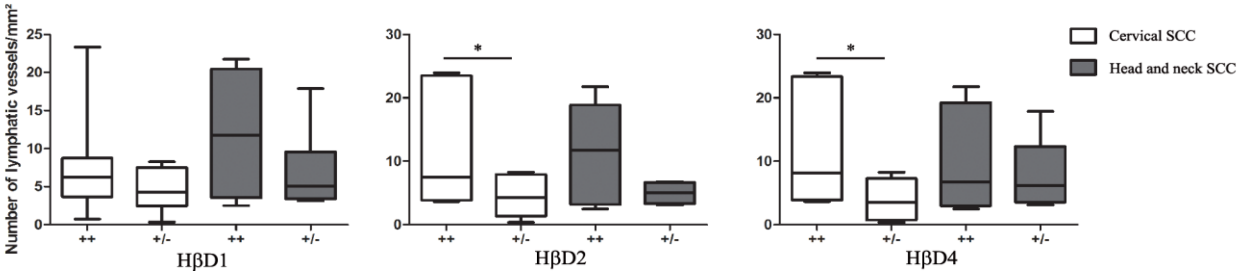


**Supplemental Figure 7.** Increased density of podoplanin$^{+}$ lymphatic vessels was detected in high HβD-expressing SCC when compared to tumors with a weakly positive HβD immunoreactivity. The tissue specimens were classified into two groups: high (score >3) or low (score ≤3) HβD1, 2 or 4 expression. The density of lymphatic vessels in tumor microenvironment was quantified by computerized image analysis and verified by manual counting.
